# Awareness regarding global warming: Popular media like films need to contribute

**DOI:** 10.4103/0019-5278.40817

**Published:** 2008-04

**Authors:** Harshal T. Pandve

**Affiliations:** Department of Community Medicine, Dr. DY Patil Medical College, Pimpri, Pune, India

Dear sir,

Global warming has emerged as one of the most important environmental issues ever to confront humanity. This concern arises from the fact that our everyday activities may be leading to changes in the earth's atmosphere that have the potential to significantly alter the planet's heat and radiation balance.[1] Recent report of intergovernmental panel on climate change (IPCC) stated that global warming is leading to rise in temperature by which is much more than expected earlier.[2]

Different sectors around the world are contributing towards creating awareness about global warming. The film media is one of the most effective as well as popular media around the world. The film media is doing constructive work of awareness regarding global warming in various parts of world. The film, “An Inconvenient Truth”, an Academy Award-winning documentary film about climate change, specifically global warming, presented by former United States Vice President Al Gore and directed by Davis Guggenheim is one of the most appreciated. Other noteworthy films include, “In Hot Water” which examines the issue of global climate changes and how it relates to the oceans. Locations include the Arctic, the Chesapeake Bay and the South Pacific islands. Ocean ecosystems are changing in ways that we are only beginning to understand. “Cooperating for Clean Air” is about Sweden's all-out assault on acid rain and global warming. Another film “Once and Future Planet” is an interesting and easy to understand explanation of what causes global warming.[3] “The 11^th^ Hour” is a documentary concerning the environmental crises caused by human actions and their impact on the planet. “The 11^th^ Hour” documents the cumulative impact of these actions upon the planet's life systems and calls for restorative action through a reshaping of human activity. “The Day After Tomorrow” is a science fiction film that depicts catastrophic effects of global warming and boasts high-end special effects, bending the lines between science, reality and science fiction.

According to IPCC report, global warming will have major impact on Asia and India is at high risk amongst the Asian countries.[2] So far, work done related to global warming is mainly confined to research, conferences, seminars and workshops. But in India, the general population has very little knowledge about the burning issue of global warming. Efforts must be taken as early as possible to create awareness about it.[4]

As in majority of the countries, in India also film media is most widely used media for entertainment. This media can be used for the purpose of creating waves of awareness about the global warming, its hazardous effects on mankind, as well as the preventive measures. Indian film industry must step forward and contribute to combat against global warming by sensitizing general population.

## References

[1] http://www.india-seminar.com/2000/486/486%20patwardhan.htm.

[2] http://www.ipcc.ch/.

[3] http://www.greenhousenet.org/global_warming_films_pop.htm.

[4] Pandve H (2007). Global warming: Need to sensitize general population. Indian J Occup Environ Med.

